# Second premolar agenesis is associated with mandibular form: a geometric morphometric analysis of mandibular cross-sections

**DOI:** 10.1038/ijos.2016.41

**Published:** 2016-11-18

**Authors:** Michael H Bertl, Kristina Bertl, Manuel Wagner, André Gahleitner, Andreas Stavropoulos, Christian Ulm, Philipp Mitteroecker

**Affiliations:** 1 Division of Orthodontics, School of Dentistry, Medical University of Vienna, Vienna, Austria; 2 Department of Periodontology, Faculty of Odontology, University of Malmö, Malmö, Sweden; 3 Division of Oral Surgery, School of Dentistry, Medical University of Vienna, Vienna, Austria; 4 Department of Diagnostic Radiology, Division of Osteoradiology, General Hospital, Medical University of Vienna, Vienna, Austria; 5 Department of Theoretical Biology, University of Vienna, Vienna, Austria

**Keywords:** agenesis, cross-sections, geometric morphometrics, mandibular form

## Abstract

The aim of this study was to compare mandibular form (i.e., size and shape) between patients with agenesis of the lower second premolar (P2) and a control group with no agenesis. Three hypotheses were tested: (H1) agenesis causes a change in mandibular morphology because of inadequate alveolar ridge development in the area of the missing tooth (mandibular plasticity); (H2) agenesis is caused by spatial limitations within the mandible (dental plasticity); and (H3) common genetic/epigenetic factors cause agenesis and affect mandibular form (pleiotropy). A geometric morphometric analysis was applied to cross-sectional images of computed tomography (CT) scans of three matched groups (*n*=50 each): (1) regularly erupted P2; (2) agenesis of P2 and the primary second molar *in situ*; and (3) agenesis of P2 and the primary second molar missing for >3 months. Cross-sections of the three areas of interest (first premolar, P2, first molar) were digitized with 23 landmarks and superimposed by a generalized Procrustes analysis. On average, the mandibular cross-sections were narrower and shorter in patients with P2 agenesis compared with that in the control group. Both agenesis groups featured a pronounced submandibular fossa. These differences extended at least one tooth beyond the agenesis-affected region. Taken together with the large interindividual variation that resulted in massively overlapping group distributions, these findings support genetic and/or epigenetic pleiotropy (H3) as the most likely origin of the observed covariation between mandibular form and odontogenesis. Clinically, reduced dimensions and greater variability of mandibular form, as well as a pronounced submandibular fossa, should be expected during the treatment planning of patients with P2 agenesis.

## Introduction

Lower second premolars (P2) are the most common congenitally missing teeth—not considering third molars—with a prevalence of 3%.^1, 2, 3, 4, 5^ Although the primary second molars may be kept in some cases,^6, 7^ more often, other treatment options are required for the replacement of the missing second premolar.^8, 9, 10^ Treatment options include autotransplantation,^11, 12^ comprehensive orthodontic treatment with either space closure^13^ or implant site development^14^ and/or an implant-supported restoration.^15, 16^ In such cases, there are specific spatial requirements for the size and cross-sectional shape of the mandibular site to ensure full bony coverage of the implant or transplanted tooth root surface.

The form of the mandible is determined by continuous bone growth and remodelling throughout ontogeny and adulthood, influenced both by genetic and epigenetic factors, such as muscle activity and biomechanical forces during mastication.^17, 18, 19, 20^ After the cessation of craniofacial growth, the alveolar ridge remains heavily influenced by the presence and position of teeth; it undergoes significant remodelling after tooth loss.^21^ Tooth agenesis is likely to affect mandibular shape, at least locally. Conversely, dental development, starting from the formation of the tooth bud, takes place within the growing mandible. Mandibular dimensions may therefore have the potential to affect tooth development, eventually leading to tooth agenesis if certain key dimensions in the mandible are below a “minimum threshold”. Indeed, it has been shown that agenesis patients differ from normal individuals in various aspects of facial morphology^22^ and that the cross-sectional dimension of the mandible covary with other facial dimensions.^23, 24^ This association of cranial and mandibular morphology with dental agenesis may also result from variation in pleiotropic genes, that is, genes that are involved both in craniofacial development and odontogenesis. Numerous syndromes and genes related to tooth agenesis have been identified,^25^ many of which also influence other craniodental traits, such as maxillary retrognathia^22, 26, 27^ and palatally displaced or transposed canines.^9, 28^

Therefore, three hypotheses can be formulated about the mechanisms linking dental agenesis to mandibular morphology:

(H1) Tooth agenesis may affect mandibular skeletal development owing to the lack of tissue growth induction (i.e., mandibular plasticity). In this case, it would be expected that differences in mandibular morphology between agenesis and non-agenesis patients would be primarily located in the vicinity of the missing tooth and other regions would remain largely unaffected.

(H2) If a minimum space within the mandible is required for successful tooth development, limited mandibular dimensions may lead to agenesis simply because of spatial limitations for the emerging tooth bud (i.e., dental plasticity). In this case, it would be expected that global mandibular morphology in agenesis patients would be considerably different compared with that in non-agenesis patients. Additionally, a minimum spatial requirement for odontogenesis should produce well-separated distributions of mandibular form between those groups.

(H3) Genetic and/or epigenetic factors affect both the mandibular form and the probability of tooth agenesis (i.e., genetic and/or epigenetic pleiotropy). Similarly to the previous hypothesis, this would also be supported by an association of tooth agenesis and global mandibular morphology. However, because of the highly polygenic basis of mandibular form,^19, 20, 29, 30^ the distributions of mandibular shape for agenesis and non-agenesis patients are likely to overlap massively.

We tested these hypotheses by comparing mandibular form (i.e., size and shape) between patients with agenesis of the lower P2 and a control group with no agenesis. To this end, we applied geometric morphometric methods to cross-sectional outlines of the mandibular body in the vicinity of P2. Geometric morphometrics, which is based on landmark coordinates instead of distance measurements, allows for the analysis and visualization complex shape differences and variance patterns.^31, 32, 33^

## Materials and methods

### Data collection

Three groups of 50 patients each, >18 years of age, with regularly erupted mandibular first premolars and first molars, were formed by screening computed tomography (CT) records of the University Clinic of Dentistry (Medical University of Vienna, Vienna, Austria) between 2004 and 2014: group 1 (“control”) consisted of patients with the lower P2 regularly erupted and *in situ*; group 2 (“agenesis”) consisting of patients with the agenesis of at least one lower P2 with the primary second molar *in situ* (if agenesis of the lower P2 was bilateral, the test side was chosen by coin toss); group 3 (“agenesis post-ex”) consisting of patients with agenesis of at least one lower P2, where the primary second molar had been extracted at least 3 months before the CT scan. Groups 1 and 3 were matched to group 2 regarding sex, age (±3 years) and mandibular side. Patients who had previously received augmentation procedures in this area were excluded.

The study protocol was approved by the ethics committee of the Medical University of Vienna (EK-No. 2005/2012).

### Dental CT scans and image preparation

**Figure 1:**
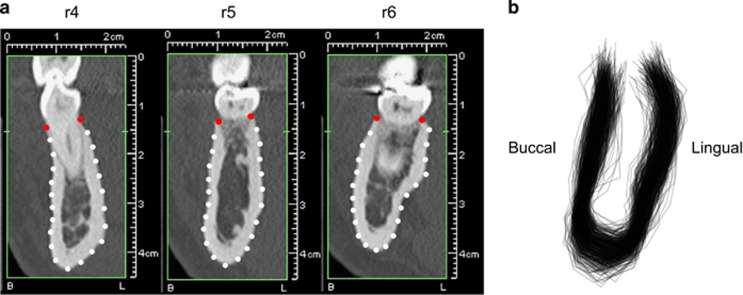
**Landmark scheme for the mandibular cross-sections.** (**a**) Cross-sectional computed tomography (CT) reconstructions at the three regions of interest: first premolar (r4), second premolar (r5) and first molar (r6) with fixed landmarks (red) and semilandmarks (white). (**b**) Reconstructed mandibular outlines of all 450 sections after standardizing the location and orientation of the landmark configurations.

Dental CT scans had been recorded with a standard dental CT investigation protocol^34^ with two different devices (Tomoscan SR-6000 (Philips Medical Systems, Eindhoven, The Netherlands) with 1 mm slice thickness, 1 mm table feed, 120 kV, 75 mA, 2 s scan time, 100–120 mm field of view, high-resolution bone filter; Somatom Sensation 4 (Siemens, Forchheim, Germany) with 0.5 mm slice thickness, 1 mm table feed, 120 kV, 80 mA, 1 s scan time, 100–120 mm field of view, high-resolution bone filter). Axial slices were used to build an orthoradial multiplanar reconstruction, which was calculated perpendicular to a manually drawn central line of the mandibular arch and to the mandibular plane. At the side of the mandible included in the analysis, three regions of interest were identified: the area of the first premolar (r4); the area of P2, the second primary molar, or the gap between the first premolar and first molar (r5); the area of the first molar (r6) (Figure 1a). For each region, a cross-sectional image was selected at either the mesiodistal midpoint of the tooth crown or at the centre of the gap between the first premolar and first molar.

All images were then arranged with the buccal side to the left and the lingual side to the right and presented in a random order to a single investigator (MW). The examiner scaled the images and identified and digitally placed the following landmarks using tpsUtil (version 1.68)^35^ and tpsDIG2 (version 2.22)^36^ software solutions. Two landmarks were placed on the buccal and lingual alveolar crest, and 21 landmarks were placed approximately equidistantly along the buccal and lingual contour of the mandible (Figure 1a).

### Morphometric analysis

The form of each mandibular cross-section was represented by the 23 landmarks, of which the two landmarks representing the buccal and lingual alveolar crest were treated as fixed anatomical landmarks and the remaining 21 as semilandmarks along the mandibular contour. After the initial, approximately equidistant placement, their exact positions were estimated by the sliding landmark algorithm to minimize the root summed squared distances (Procrustes distance) among all superimposed cross-sections and their sample average.^37, 38^ The resulting 450 configurations of 23 landmarks were superimposed by a Generalized Procrustes Analysis^33, 39^ (Figure 1b). This least squares-based superimposition standardizes the landmark configurations for overall position, scale and orientation, yielding a set of shape coordinates for each cross-section. For the present study, not only the shape of the cross-sections but also their size was of interest. Thus, for certain analyses, the shape coordinates were rescaled by the configuration's centroid size to perform the analyses of the full form information (i.e., size and shape).^40, 41^ Centroid size is a standard measure of the overall size of a landmark configuration; it equals the square root of the summed squared distances of the landmarks from their centroid (average landmark position).

Group-wise means and variances of the cross-sectional area (based on the polygon defined by the measured landmarks) and centroid size, as well as the total variance of cross-sectional shape were calculated. The group mean forms, together with extrapolated versions, were represented as reconstructed cross-sectional forms. The differences between the group mean forms were analysed by a principal component analysis (PCA) of the average rescaled shape coordinates. The statistical significance of group mean differences was estimated by permutation tests,^42^ based on the Euclidean distance between-group mean forms as the test statistic and 10 000 random permutations.

To represent the individual variation around these means, the individual configurations were projected onto the two principal components (PCs) of the mean forms (between-group PCA).^43^ In addition, scores (orthogonal projections) for each individual along the mean difference vectors were computed. These scores represent the individual expression of the form features that differ most between the groups. Histograms of these scores allowed for the assessment of separation or overlap among groups.

## Results

Each group comprised 19 male and 31 female patients. In 24 of these patients, the left side of the mandible was measured, and in 26, the right side. The mean ages of the control, agenesis and agenesis post-ex groups were 22.9±5.5, 22.8±5.6 and 25.0±8.1 years, respectively.

**Figure 2:**
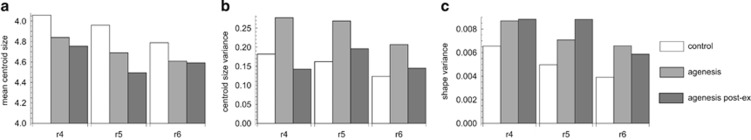
**Mean centroid size (a), variance of centroid size (b) and total shape variance (c) of the three groups and the three dental regions (r4, r5 and r6)**.

The cross-sectional area was significantly smaller in the two agenesis groups compared with that in the control group (*P*<0.005 for both r4 and r5; *P*=0.02 for r6; Table 1). Similarly, centroid size, as a measure of overall size, was smaller in both agenesis groups compared with the control group for the three regions of interest (Figure 2). In contrast, the variance of both the centroid size and shape of the mandibular cross-section was smallest in the control group (Figure 2). Differences in the variance of the cross-sectional area were not as pronounced as those for centroid size and shape. However, the corresponding coefficients of variation for cross-sectional area (standard deviation divided by the mean as a size correction) were clearly the smallest in the control group.

**Figure 3:**
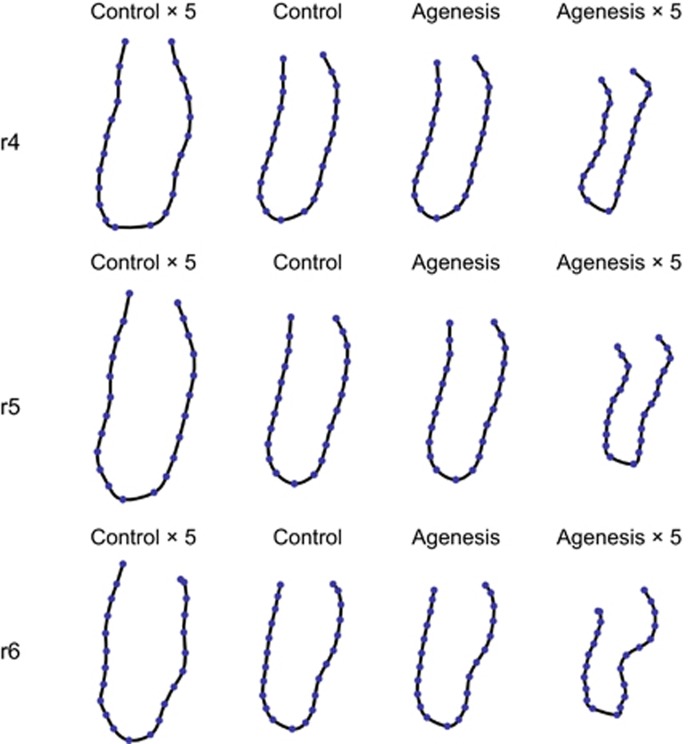
**Mean forms of the control group and the agenesis group for all three regions (r4, r5 and r6), together with fivefold extrapolations of these group differences**. For example, to compute to the upper right configuration—the extrapolated agenesis form—five times the average difference between agenesis and control group was added to the average control form.

**Figure 4:**
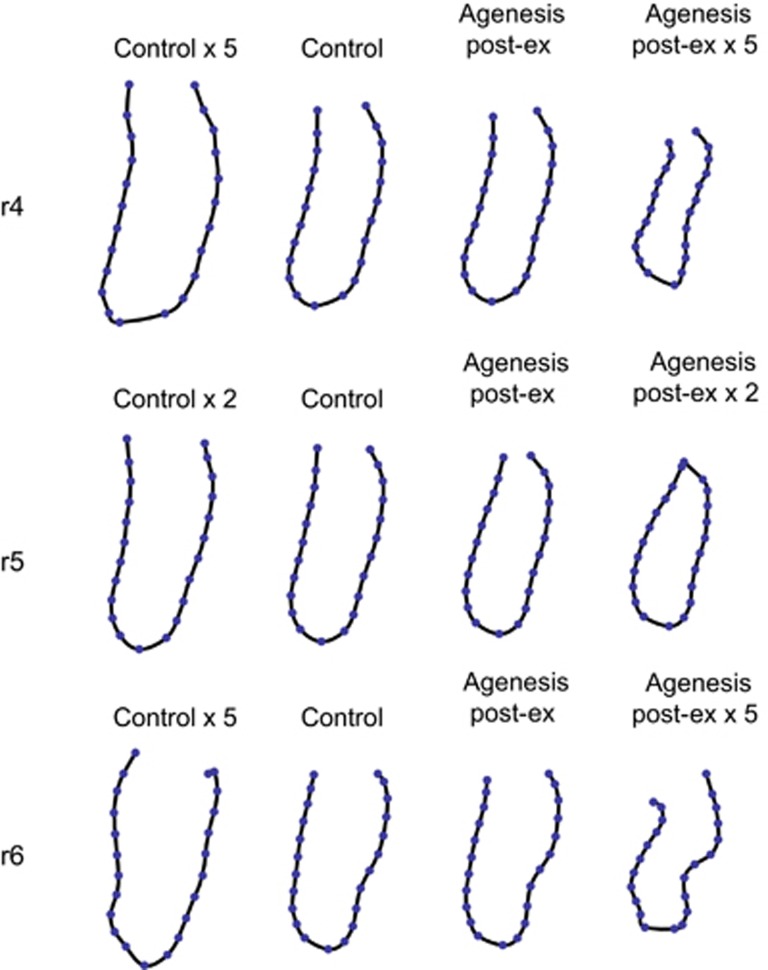
**Mean forms of the control group and the agenesis post-ex group for all three regions (r4, r5 and r6), together with fivefold extrapolations of these group differences.**

Apart from these size differences, the mandibular cross-sections also differed in shape between the agenesis groups and the control group. Although the average differences were subtle, they were consistent across both agenesis groups and across the three regions of interest (*P*=0.004 for r4 and *P*=0.02 for both r5 and r6). Figures 3 and 4 show the group mean forms along with extrapolations of their mutual differences. On average, the three regions of interest were narrower and shorter in the agenesis groups compared with those in the control group. Furthermore, in patients with dental agenesis the mandibular cross-sections featured a pronounced lingual alveolar plate and a distinct concavity (submandibular fossa) underneath the mylohyoid line. The extent of this concavity increased from the r4 to the r6 region.

**Figure 5:**
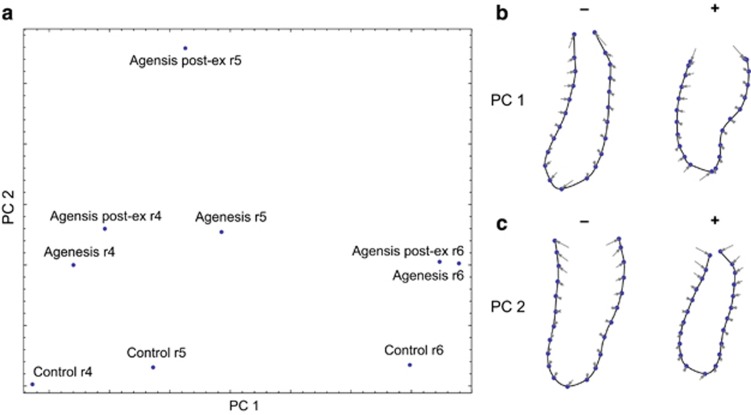
**Principal component analysis (PCA) of mandibular form.** (**a**) Scatterplot of the first two principal components (PCs) of the group mean forms. (**b**, **c**) Visualization of the form differences associated with the two PCs.

The consistent pattern of form differences is also shown by the PCA in Figure 5. Whereas PC 1 mainly contrasts the three analysed dental regions, PC 2 similarly distinguishes the control group from the agenesis patients for all three regions of interest. Extraction of the primary molar at the r5 region in the post-ex group induced more pronounced shape differences compared with the other groups; thus, the corresponding mean form deviated from the other agenesis groups in the PC plot.

**Figure 6:**
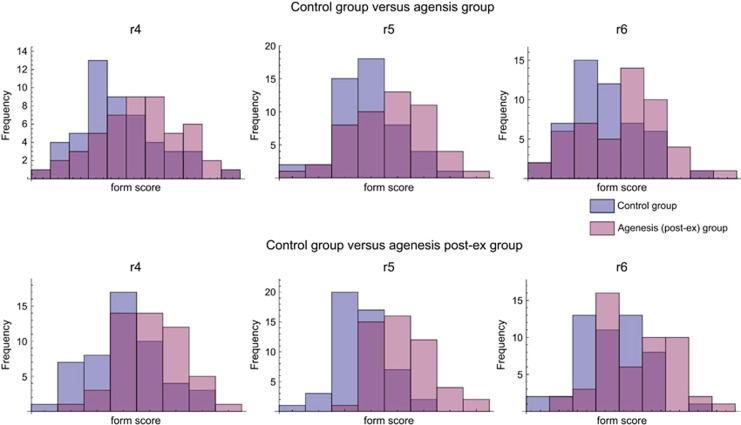
**Histograms of individual scores for the form features that differ most between the control and agenesis groups (upper panel) and between the control and agenesis post-ex group (lower panel)**. These scores are orthogonal projections of the rescaled shape coordinates on the mean difference vectors.

Despite the clear pattern of average form differences, individual variation around these means was substantial, leading to a massive overlap of group distributions. To display this group overlap, Figure 6 presents histograms of individual form scores that maximize the average difference between the control and agenesis groups. Despite this maximization of group differences, the form scores show strong overlap for all group comparisons. Similarly, the between-group PCA of all individuals in Supplementary Figure S1 shows a strong multivariate overlap among the groups.

## Discussion

The results of this study clearly showed that the cross-sectional mandibular form (shape and size) in patients with agenesis of the lower P2 differed significantly from that of patients without agenesis of P2. These differences were apparent not only in the region of P2 (r5) but also in the neighbouring regions of the first premolar (r4) and first molar (r6). Mandibular form differences extending (at least one tooth) beyond the agenesis area are not consistent with H1, the hypothesis of mandibular plasticity, which predicted that skeletal differences are constrained to the close vicinity of the affected region (r5). Only the alveolar crest reacted locally to deciduous tooth loss (compare the pronounced shape differences at r5 between the control and agenesis post-ex groups in Figures 4 and 5). It has been shown previously that extraction of the primary molar in agenesis patients results in a 25% reduction of the original alveolar ridge width within 3 years and a 30% reduction within 6 years.^44^

Despite the average form differences, individual shape distributions greatly overlapped between both agenesis groups and the control group. This finding favours H3, genetic or epigenetic pleiotropy, over H2, the hypothesis of dental plasticity. If a consistent minimum of mandibular dimensions acts as a threshold for dental development, the mandibular form variations would be more clearly separated between agenesis and control groups. The strong overlap that we found could to some degree result from a threshold that is highly variable across individuals. It seems more likely, however, that genetic or epigenetic pleiotropic factors influence both mandibular form and odontogenesis. Such pleiotropic factors would account for the association between tooth agenesis and average mandibular form, whereas the highly polygenic basis of both traits would explain the considerable individual variation and the group overlap of mandibular form distributions.

The genetic network underlying tooth agenesis has received increased attention in the recent literature. Multiple signalling pathways important to tooth development as well as more than 150 syndromes and 80 genes related to tooth agenesis have been identified todate.^25^ The homeobox gene *MSX1*, which controls proliferation and differentiation in a variety of cell types, is one of the many candidate genes underlying agenesis. Several MSX1 mutations have been identified in tooth agenesis patients, and MSX1 is also a direct downstream target of WNT/β-catenin signalling during craniofacial development. Imbalances in these signalling interactions may account for failures in both craniofacial development and odontogenesis.^25, 45^ A pleiotropic genetic basis of tooth agenesis is also supported by the frequent co-occurrence with other dental anomalies, such as palatally displaced or transposed canines.^9, 28^

A polygenic basis of mandibular morphology with numerous pleiotropic effects has been documented extensively in mice.^19, 20, 29, 46^ Cheverud *et al.*^29^ identified 26 chromosomal regions that affected more than two mandibular traits, half of which were related to the tooth-baring alveolar region. Workman *et al.*^20^ found that many of these traits also relate to molar morphology. In humans, maxillary retrognathia has been associated with mild^22, 27^ and severe^26^ forms of maxillary tooth agenesis. Similarly, mandibular retrognathia was prevalent in patients with mandibular tooth agenesis, suggesting an impeded sagittal development of the jaw associated with tooth agenesis.^22^ Vertical relations appear less affected.^22, 47^ Similar to our results, these studies report standard deviations in the agenesis groups that markedly and consistently exceed those of individuals with normal dental development. Such a destabilization of development by genetic or environmental perturbations, leading to increased phenotypic variability, has been documented in various biological and biomedical contexts.^48, 49, 50, 51, 52, 53^

The cross-sectional form of the human mandible has been studied in anatomical sections^54^ as well as CT scans,^24, 55, 56^ and was associated with varying facial dimensions^24, 55^ and dental inclinations.^56^ The submandibular fossa was previously found to increase from the premolar to the molar region, ranging from 0 to 5 mm in depth,^57^ which is reflected by PC 1 in our study. The extent of this fossa has been described as highly variable.^57^ While we found the submandibular fossa to increase in depth from r4 to r6, the variance of both size and shape decreased from r4 to r6 in the control group (Figure 2). It has been previously shown that cross-sectional dimensions of the mandible are sexually dimorphic;^24^ indeed, we also found sexual differences in cross-sectional size and shape that were consistent across regions of interest and agenesis groups. Therefore, these differences did not confound our results on agenesis despite the unequal number of males and females herein, which simply represents the higher prevalence of tooth agenesis in females.^3, 47^

Our findings of reduced mandibular dimensions in agenesis patients have clinical implications for the restoration of congenitally missing P2 by endosseous replacement (tooth autotransplantation or implant installation) with specific minimum bone volume requirements.^58^ The shape differences between agenesis and non-agenesis patients—particularly the prevalence of a pronounced submandibular fossa in agenesis patients—may pose a risk for lingual perforation during tooth extraction or implantation. Early reports of such perforations due to extractions date back more than half a century,^59, 60^ whereas more recently, similar complications have been reported for the placement of dental implants.^61, 62, 63^ A submandibular fossa depth >2 mm has been identified as a risk factor for lingual cortex perforation during implant placement,^64^ putting 10%–18% of patients at risk when installing a 10-mm implant.^57, 65^ Penetration of the lingual plate in the submandibular fossa endangers the Nevus mylohyoideus, the A. and V. mylohyoidea, the glandula submandibularis, and the M. mylohyoideus. Because the submandibular fossa depth is often difficult to estimate by clinical examination, from only the alveolar bone width, or by standard two-dimensional imaging,^62, 64^ three-dimensional radiographic examination of the posterior mandible is indicated for the treatment planning of agenesis patients.

## Conclusions

1. Cross-sectional mandibular size and shape differ significantly between patients with and without agenesis of the lower P2.
2. This effect extends beyond a localized morphological change, suggesting pleiotropic genetic/epigenetic effects as mechanisms that link craniofacial growth and odontogenesis.
3. Clinically, a greater variability in mandibular form, a more pronounced lingual alveolar plate, and a distinct submandibular fossa underneath the mylohyoid line should be expected when treating patients with lower P2 agenesis.

## Figures and Tables

**Table 1:** Means and standard deviation of the mandibular cross-sectional areas (in cm^2^) at the three regions of interest (r4, r5, r6) for the control group and the two agenesis groups

Groups	r4	r5	r6
Control	2.68 (0.47)	2.65 (0.42)	2.72 (0.37)
Agenesis	2.41 (0.50)	2.37 (0.50)	2.52 (0.49)
Agenesis post-ex	2.35 (0.44)	2.11 (0.45)	2.52 (0.46)
