# Supplementary figures and images for: Second premolar agenesis is associated with mandibular form: a geometric morphometric analysis of mandibular cross-sections
Source: Int J Oral Sci. 2016 Nov 18;8(4):254–60. doi: 10.1038/ijos.2016.41 (PMC5168418; doi:10.1038/ijos.2016.41)

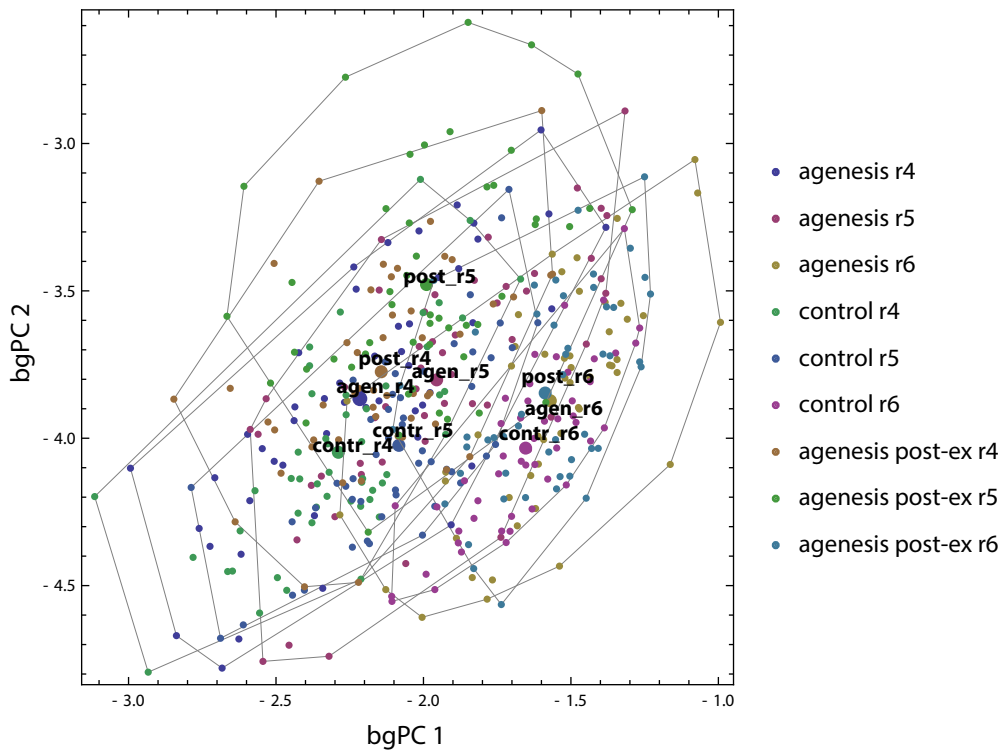

Supplement: Supplementary Figure S1 [file ijos201641x1.pdf]
